# Strain-Level Analysis of *Bifidobacterium* spp. from Gut Microbiomes of Adults with Differing Lactase Persistence Genotypes

**DOI:** 10.1128/mSystems.00911-20

**Published:** 2020-09-29

**Authors:** Victor Schmidt, Hagay Enav, Timothy D. Spector, Nicholas D. Youngblut, Ruth E. Ley

**Affiliations:** a Department of Microbiome Science, Max Planck Institute for Developmental Biology, Tübingen, Germany; b Department of Twin Research & Genetic Epidemiology, King’s College London, London, United Kingdom; University of California San Diego

**Keywords:** *Bifidobacterium*, lactase persistence, microbiome, strain stability, gut microbiome, human microbiome

## Abstract

One of the strongest associations between human genetics and the gut microbiome is a greater relative abundance of *Bifidobacterium* in adults with lactase gene (*LCT*) single nucleotide polymorphisms (SNPs) associated with lactase nonpersistence (GG genotypes), versus lactase persistence (AA/AG genotypes). To gain a finer-grained phylogenetic resolution of this association, we interrogated 1,680 16S rRNA libraries and 245 metagenomes from gut microbiomes of adults with various lactase persistence genotypes. We further employed a novel genome-capture-based enrichment of *Bifidobacterium* DNA from a subset of these metagenomes, including monozygotic (MZ) twin pairs, each sampled 2 or 3 times. B. adolescentis and B. longum were the most abundant *Bifidobacterium* species regardless of host *LCT* genotype. *LCT* genotypes could not be discriminated based on relative abundances of *Bifidobacterium* species or *Bifidobacterium* community structure. Three distinct metagenomic analysis methods of *Bifidobacterium*-enriched DNA revealed intraindividual temporal stability of B. longum, *B. adolescentis*, and B. bifidum strains against the background of a changeable microbiome. Two of our three methods also observed greater strain sharing within MZ twin pairs than within unrelated individuals for *B. adolescentis*, while no method revealed an effect of host *LCT* genotype on *Bifidobacterium* strain composition. Our results support a “rising tide lifts all boats” model for the dominant bifidobacteria in the adult gut: their higher abundance in lactase-nonpersistent than in lactase-persistent individuals results from an expansion at the genus level. *Bifidobacterium* species are known to be transmitted from mother to child and stable within individuals in infancy and childhood: our results extend this stability into adulthood.

**IMPORTANCE** When humans domesticated animals, some adapted genetically to digest milk into adulthood (lactase persistence). The gut microbiomes of people with lactase-persistent genotypes (AA or AG) differ from those with lactase-nonpersistent genotypes (GG) by containing fewer bacteria belonging to the bifidobacteria, a group which contains beneficial species. Here, we asked if the gut microbiomes of adults with GG and AA/AG genotypes differ in the species of bifidobacteria present. In particular, we used a novel technique which allowed us to compare bifidobacteria in adults at the strain level, without the traditional need for culturing. Our results show that the GG genotype enhances the abundance of bifidobacteria regardless of species. We also noted that a person’s specific strains are recoverable several years later, and twins can share the same ones. Given that bifidobacteria are inherited from mother to child, strain stability over time in adulthood suggests long-term, multigenerational inheritance.

## Abstract (precis)

When humans domesticated animals, some adapted genetically to digest milk into adulthood (lactase persistence). The gut microbiomes of people with lactase-persistent genotypes (AA or AG) differ from those with lactase-nonpersistent genotypes (GG) by containing fewer bacteria belonging to the bifidobacteria, a group which contains beneficial species. Here, we asked if the gut microbiomes of adults with GG and AA/AG genotypes differ in the species of bifidobacteria present. In particular, we used a novel technique which allowed us to compare bifidobacteria in adults at the strain level, without the traditional need for culturing. Our results show that the GG genotype enhances the abundance of bifidobacteria regardless of species. We also noted that a person’s specific strains are recoverable several years later, and twins can share the same ones. Given that bifidobacteria are inherited from mother to child, strain stability over time in adulthood suggests long-term, multigenerational inheritance.

## INTRODUCTION

Lactose tolerance arose in European, African, and Middle Eastern human populations with animal domestication (1–3). The genetic underpinnings of lactose tolerance represent one of the strongest signals of recent selection in the human genome (3). The enzyme lactase metabolizes lactose, the primary carbohydrate in mammalian milk. The gene regulatory region of the lactase gene (*LCT*) controls the downregulation of lactase after weaning (4). Allelic variants that inhibit downregulation, resulting in lactase persistence, occur in an estimated 35% of humans (1). Lactase persistence allows hydrolyzation of lactose and uptake of the resulting glucose and galactose directly in the small intestine of adults and is linked to lactose tolerance.

In a striking parallel, one of the strongest signals for human genotype effects on the gut microbiome also relates to lactase persistence. In Western populations, individuals with a lactase-persistent genotype harbor a significantly lower relative abundance of *Bifidobacterium* in their gut microbiomes than do nonpersistent individuals (5–8). The association was found to be stronger when dairy consumption in the lactase-nonpersistent individuals was considered (9). Together, these observations suggest that for lactase-nonpersistent individuals, *Bifidobacterium* may benefit from the availability of lactose in the gut. *Bifidobacterium* is a large genus whose members are often metabolic specialists with regard to particular nutrients (10), which suggests that not all *Bifidobacterium* species may respond equally to lactose availability. However, beyond an overall enrichment of the *Bifidobacterium* genus, the effects of the lactase persistence genotype on the bifidobacterial community in the gut remain unclear.

Here, we aimed to interrogate the *LCT*-*Bifidobacterium* link at a finer phylogenetic resolution. We reexamined public metagenomic (11) and 16S rRNA gene data (5) from a UK twin cohort with both lactase-persistent (AA, AG) and nonpersistent (GG) individuals. We then performed genome-capture enrichment of *Bifidobacterium* from 11 twin pairs of each genotype across two or three time points per individual and sequenced each metagenome before and after genome capture. With these data, we asked if lactase persistence genotype influenced strain composition, longitudinal stability within an individual, or similarity within families for three species (B. longum, B. adolescentis, and B. bifidum). Our results suggest a proportional increase of the predominant *Bifidobacterium* species in the gut microbiomes of the lactase-persistent compared to nonpersistent individuals. We observed strong strain stability within individuals, and sharing of some strains between MZ twins, independent of *LCT* genotype group.

## RESULTS

**FIG 1:**
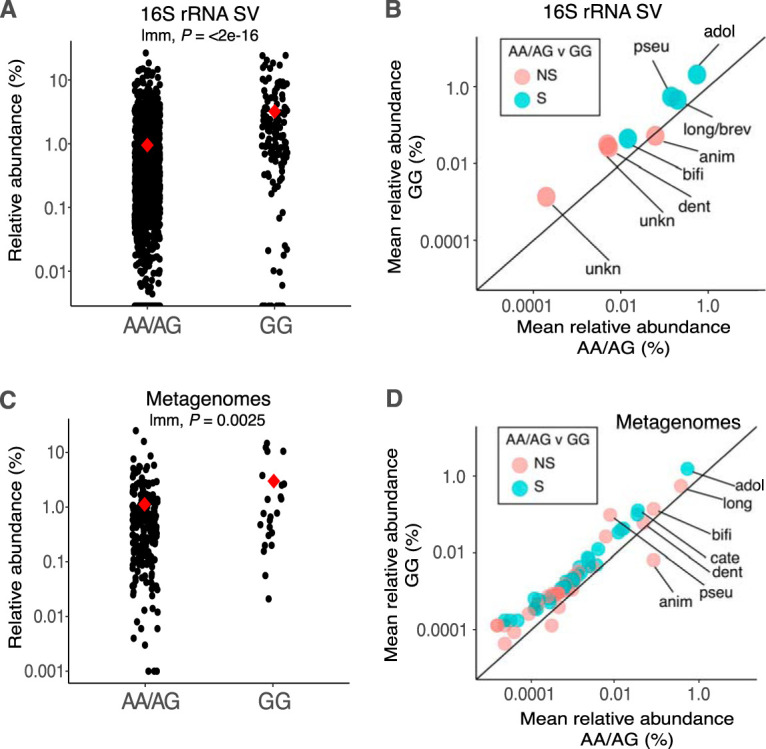
Lactase-persistent genotype enriches most human associated *Bifidobacterium* species. (A) Relative abundance of reads annotated as *Bifidobacterium* at the genus level from lactase-persistent (AA/AG) and nonpersistent (GG) individuals based on 16S rRNA SV taxonomic annotations (*n*: AA/AG = 1,549, GG = 131). (B) Mean relative abundance of *Bifidobacterium* species in AA/AG (*x* axis) and GG (*y* axis) individuals based on 16S rRNA SV taxonomic annotations. Colors indicate significant enrichment in GG. The 1:1 line designates equal proportion in each genotype, and points above the line therefore indicate enrichment in GG and vice versa. Taxa that occurred in only one genotype are not shown. Species are *B. adolescentis* (adol), B. longum (long), *B. pseudocatenulatum* (pseu), *B. animalis* (anim), *B. bifidum* (bifi), *B. dentium* (dent), and *B. catenulatum* (cate; appears only in panel D). Unknown (unkn) indicates SVs that cannot be resolved beyond genus level. NS/S represents nonsignificant or significant enrichment in GG, respectively, according to bootstrapped Wilcoxon rank sum tests. (C) Relative abundance of reads annotated as *Bifidobacterium* at the genus level from lactase-persistent (AA/AG) and nonpersistent (GG) individuals’ metagenomic annotations (*n*: AA/AG = 222, GG = 23). Red diamonds indicate means and *P* values from linear mixed models. (D) Mean relative abundance of *Bifidobacterium* species in AA/AG (*x* axis) and GG (*y* axis) individuals as revealed by metagenomic annotations. For clarity, only the 7 most abundant taxa with species-level annotations are labeled (full list available in Table S1). Colors are as in panel B.

Our analysis of 16S rRNA gene sequence variants (SVs) confirmed our previous operational taxonomic unit (OTU)-based report (5) of significantly greater mean relative abundance of *Bifidobacterium* in lactase-nonpersistent (GG) versus persistent (AA/AG) individuals (mean AA/AG = 0.96% ± 0.05% and mean GG = 3.22% ± 0.4% [standard error {SE}], linear mixed-model *P* < 4.5e^−16^) (Fig. 1A; see also Table S1, tab 1, in the supplemental material). This analysis revealed 13 *Bifidobacterium* SVs which occurred in at least two of 1,680 samples. Five of the 13 SVs were unambiguously identified to the species level, while two fell within a broader B. longum*/*B. breve clade. Both host genotype groups (GG versus AA/AG) were dominated, in order of abundance, by *B. adolescentis*, B. longum*/breve*, B. pseudocatenulatum, B. animalis, and *B. bifidum*. Together these taxa represented over 98.6% of all sequences assigned to the *Bifidobacterium* genus, although only 1.1% of all sequences across all taxa (Table S1, tab 1). These five species had similar proportions of the total *Bifidobacterium* community in both genotype groups and almost identical rank orders (GG, 93%; AA/AG, 91%) (Fig. 1B; Table S1, tab 1). Based on SVs, *B. animalis* and B. dentium were the only taxa with species-level designations that did not have significantly greater relative abundance in GG than in AA/AG genotypes.

10.1128/mSystems.00911-20.8 TABLE S1 (Tab 1) Taxonomic annotations of 16S rRNA SVs (top, *n* = 1,680) and metagenomic sequences (bottom, *n* = 245). (Tab 2) Overview of participant data. (Tab 3) Reference genomes used in the capture array. (Tab 4) Details of StrainPhlAn multilocus sequence alignments for each of the species considered. (Tab 5) Summary of strain-level methods used in the paper and an extension of Table 1 and Table 2 to include *P* values of significant tests. (Tab 6) *P* values and comparison numbers for the differences in mean synteny scores between pairwise comparison categories, for each species broken down by genomic region and in aggregate. (Tab 7A) E. coli genomes used for the validation of the synteny-based method. (Tab 7B) Genomic regions in the E. coli genome used for calculation of pairwise synteny scores. Download Table S1, XLSX file, 0.03 MB. Copyright © 2020 Schmidt et al. 2020 Schmidt et al. This content is distributed under the terms of the Creative Commons Attribution 4.0 International license.

After normalization of *Bifidobacterium* within each genotype (thereby removing the effect of an overall enrichment of the genus), discriminant analysis using linear discriminant analysis effect size (LEfSe) (12) revealed no discriminant *Bifidobacterium* SVs between GG and AA/AG individuals (data not shown). This result indicates similar proportional abundances of each *Bifidobacterium* species within each host genotype group, despite an overall greater relative abundance of all taxa in GG versus AA/AG genotypes. Analysis of similarity (ANOSIM) permutation tests on the Bray-Curtis Dissimilarity (BCD) matrix of within-genotype normalized *Bifidobacterium* SV matrices also revealed no significant community clustering by host genotype group (ANOSIM *P* > 0.05), further supporting a proportional increase across most taxa rather than genotypic selection of specific species or strains within the genus.

We also assessed the association between frequency of dairy consumption and *Bifidobacterium* SVs for the 783 samples for which both data sets were accessible. Linear mixed-effect models, with genotype as a random variable, revealed no overall association between dairy consumption and the relative abundance of the genus (linear mixed model *P* = 0.113). When SVs were interrogated independently, *B. animalis* and an unclassifiable bifidobacterium were the only two SVs which showed significant associations (*B. animalis*, *P* = 0.0023; *B. unknown1*, *P* = 0.014). Interestingly, when each genotype was considered independently, significant associations with levels of dairy consumption were observed for *B. animalis* within GG individuals, but not in AA/AG individuals (generalized linear model: GG, *P* = 0.03; AA/AG, *P* = 0.13).

Our taxonomic annotations of metagenomic reads from existing TwinsUK metagenomes (11) revealed an overall enrichment of the *Bifidobacterium* genus in lactase-nonpersistent individuals (mean AA/AG = 1.1% ± 0.15% and mean GG = 2.8% ± 0.9%, linear mixed model *P* = 0.0025) (Fig. 1C; Table S1, tab 1) and a proportional increase across most *Bifidobacterium* species in GG versus AA/AG individuals (Fig. 1D). Largely concordant with the results of the 16S rRNA SV analysis, *Bifidobacterium* metagenome annotations from both host genotype groups were dominated by *B. adolescentis*, B. longum, *B. bifidum*, and *B. animalis*, which together represented more than 80% of all *Bifidobacterium* sequences, though only 1% of the overall community (Fig. 1D; Table S1, tab 1). Taxonomic annotations from metagenomes showed a greater diversity of *Bifidobacterium* taxa across the full data set compared to the 16S rRNA gene SV-based analysis, despite a lower sample count (245 metagenomes versus 1,680 16S rRNA samples), with 65 species and subdivisions identified (versus 13 SVs).

Functional annotations of the 245 metagenomes revealed no MetaCyc *Bifidobacterium* metabolic pathways with significantly different relative abundances between the two genotype groups (pairwise Kruskal-Wallis H-tests for mean abundance of each *Bifidobacterium* pathway in GG versus AA/AG individuals, Bonferroni multiple-comparison correction, implemented in HUMAnN2; data not shown). However, low or zero read coverage for most *Bifidobacterium* pathways in the metagenomes limited the power to assess differences between host genotype groups at the functional level.

### Genome capture enriches *Bifidobacterium* DNA.

**FIG 2:**
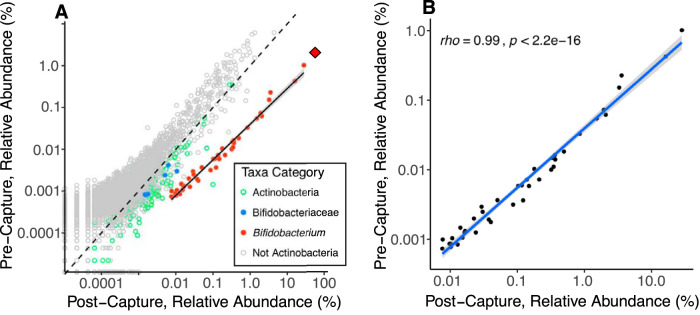
*Bifidobacterium* is proportionally enriched using genome capture. (A) Relative abundance of all species-level annotations from the genome capture subset (*n* = 46), with each point representing the mean relative abundance of a distinct species-level classification before (precapture) and after (postcapture) the *Bifidobacterium*-DNA capture assay. Points are colored by taxonomic relatedness to *Bifidobacterium.* Pre- and postcapture libraries from a single sample were shotgun sequenced independently. The dashed 1:1 line represents equal relative abundances in pre- and postcapture libraries, and points below the 1:1 line are enriched in postcapture libraries relative to precapture libraries. The solid black line shows the least-squares regression between pre- and postcapture for the *Bifidobacterium* annotations. The shallower slope of the *Bifidobacterium* enrichment line than of the 1:1 line indicates greater enrichment of more common taxa. The red diamond shows the total relative abundance of *Bifidobacterium* (i.e., the sum of all *Bifidobacterium* annotations) in each of the pre- and postcapture libraries. (B) Same data as plot A but showing only *Bifidobacterium* annotations. The blue line shows least-squares regression with 95% confidence intervals; Spearman’s rho is shown along with the significance of the correlation.

We used genome capture (13, 14) to enrich for *Bifidobacterium* DNA in metagenome libraries generated from 22 individuals each with 2 or 3 samples obtained at different time points (46 samples total representing 11 GG and 11 AA individuals). Each genotype group also included 4 sets of adult monozygotic twin siblings (Table S1, tab 2). Our custom genome-capture array included a total capture space of >94 Mb and 89k capture targets built from 47 reference *Bifidobacterium* genomes spanning the entire diversity of the genus (Table S1, tab 3). The capture reaction was largely genus specific, with low levels of enrichment of non-*Bifidobacterium Bifidobacteriaceae* and other non-*Bifidobacterium Actinobacteria* (Fig. 2A). The capture reaction increased the mean relative abundance of all bifidobacteria across our metagenomic sample subset from 2.1% (±0.27% [SE]) to 60.2% (±3.7% SE]), representing a nearly 30-fold increase from pre- to postcapture libraries. The mean relative abundance of sequences annotated as a specific *Bifidobacterium* taxon across all postcapture libraries was proportional to that taxon’s initial relative abundance in the precapture libraries (*rho *= 0.99, *P* < 2.2e^−16^, Fig. 2B), and the mean relative abundance ranking order of the top 5 taxa was the same in pre- and postcapture libraries (data not shown). We note that no analysis method presented below uses post-genome-capture data to make conclusions regarding abundance or relative abundance, given the uncertainties of differential capture between species. Only sequence-composition-based analyses are used (e.g., multilocus sequence alignments [MLSAs], marker single nucleotide polymorphisms [SNPs], and synteny) from postcapture data.

### Strain-level analysis of *Bifidobacterium* spp. in postcapture metagenomes.

We compared strains recovered from postcapture metagenomes using three methods: (i) we generated genome-wide strain phylogenies using multilocus sequence alignments (MLSAs) via StrainPhlAn (15), (ii) we used a marker-SNP strain tracking analysis implemented in the Metagenomic Intra-Species Diversity Analysis System (MIDAS) (16), and (iii) we used a custom synteny-based approach. We compared strains in all possible pairwise sample comparisons from our genome-capture subset (*n* = 46). We then grouped each comparison into one of three categories: intraindividual (comparison between samples from the same person over time), same family (between monozygotic twins within a twinship), and unrelated (between samples from unrelated individuals). These categories allowed us to assess strain stability through time within an individual (intraindividual comparisons) and the influence of monozygotic twins (same-family comparisons). Our postcapture libraries yielded sufficient reads to conduct these analyses in four species, B. longum, *B. adolescentis*, *B. animalis*, and *B. bifidum*, with sample numbers shown in Table 1.

**TABLE 1:** Sample number and comparison category overview for StrainPhlAn and MIDAS strain-level analyses

	StrainPhlAn				MIDAS			
	No. of samples with sufficient coverage for phylogeny	No. of comparisons			No. of samples with sufficient coverage for marker-SNP analysis	No. of comparisons		
		Intraindividual	Same family	Unrelated		Intraindividual	Same family	Unrelated
*B. adolescentis*	43	23	37	843	37	17	30	619
B. longum	39	20	29	692	36	15	24	591
*B. bifidum*	19	8	8	155	20	6	8	176
*B. animalis*	11	3	2	50	15	4	3	98

Total		54	76	1,740		42	65	1,484

### MLSAs showed greater similarities of strain sequences in intraindividual than in unrelated comparisons.

Using StrainPhlAn, we created MLSAs across nearly 200 strain-resolving marker genes derived from a species’ core genome (genes shared by all strains in the species). Of the postcapture shotgun metagenomes, *B. adolescentis*, B. longum, *B. bifidum*, and *B. animalis* had sufficient coverage for 43, 39, 19, and 11 samples, respectively (minimum of 2× coverage across entire alignment) (Table 1). The marker gene MLSAs ranged from 36 to 83 kb in length depending on species (Table S1, tab 4). The mean number of polymorphic sites within a sample across an alignment ranged from 0.39% to 0.92%, while the dominant allele at each site ranged upward from 76%, suggesting low strain diversity within samples (Table S1, tab 4, and Fig. S1).

10.1128/mSystems.00911-20.1 FIG S1 StrainPhlAn alignment statistics show low intrasample strain diversity. (A) The mean percentage of polymorphic sites along our MLSA for each of the four species considered. (B) The mean dominant allele frequency across all polymorphic sites within an MLSA. Boxplots show median and quartiles. Species: adol, *B. adolescentis*; anim, *B. animalis*; bifm, *B. bifidum*; long, B. longum. Significance levels are Wilcoxon rank sum tests. *, *P* ≤ 0.05; **, *P* ≤ 0.01; ***, *P* ≤ 0.001; ****, *P* ≤ 0.0001. Nonsignificant comparisons are not indicated. Download FIG S1, PDF file, 0.04 MB. Copyright © 2020 Schmidt et al. 2020 Schmidt et al. This content is distributed under the terms of the Creative Commons Attribution 4.0 International license.

**FIG 3:**
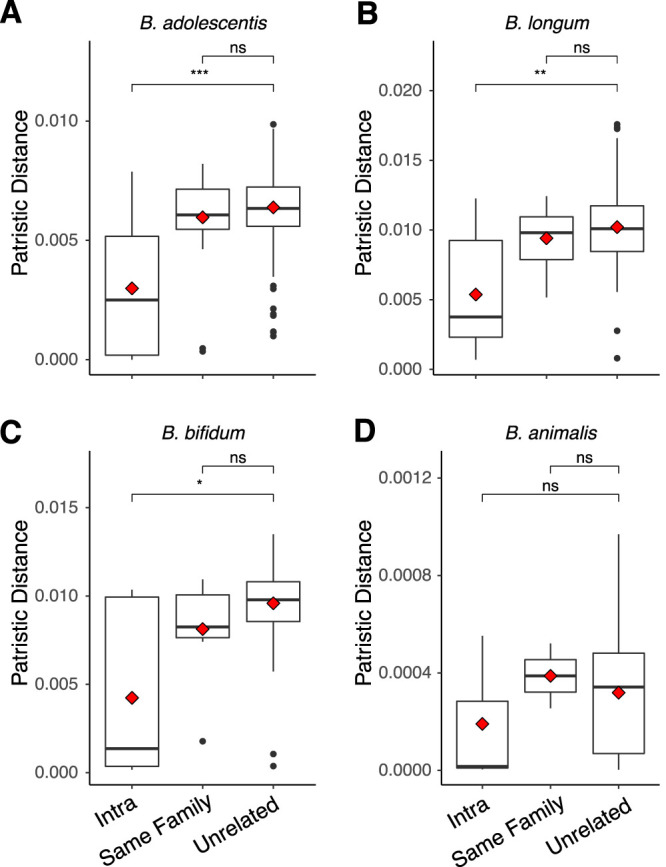
Patristic distances between samples from RAxML phylogenies of four strains show intraindividual strain similarity. Patristic distances (branch lengths) between all samples within our StrainPhlAn phylogenies, with each point representing the distance between two samples on a single phylogeny. Distances are separated by the category of comparison: intraindividual (same person over time), same family (comparisons between twin siblings), and unrelated (comparisons between unrelated people). Species are *B. adolescentis* (A), B. longum (B), *B. bifidum* (C), and *B. animalis* (D). Boxplots show median and quartiles, while red diamonds show means. Significance levels are median Wilcoxon rank sum tests after 999 bootstraps to smallest group size. ns, *P* > 0.05; *, *P* ≤ 0.05; **, *P* ≤ 0.01; ***, *P* ≤ 0.001; ****, *P* ≤ 0.0001.

**FIG 4:**
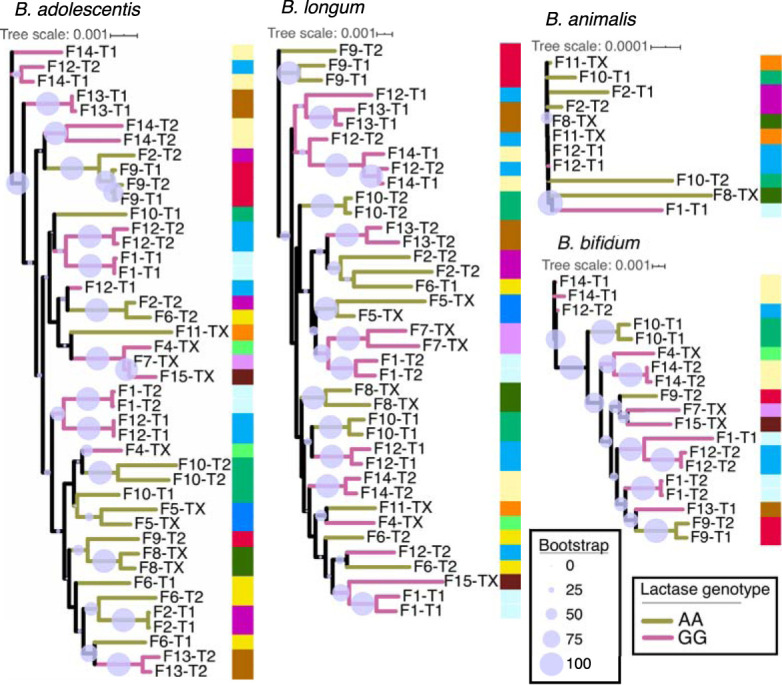
Strain phylogenies show longitudinal intraindividual strain similarities. RAxML phylogenies of StrainPhlAn MLSAs for each of the four species considered. Tree branches are colored by lactase persistence genotype. Columns of colors at each node reference family IDs, with multiple samples from the same individual and their twin having the same color. Samples are labeled by family ID, then by twin ID within the family. Identical labels imply samples from different time points of the same individual (i.e., intraindividual sample). Twin IDs marked with “X” imply that only a single twin had representation on the tree or that the individual had no twin in the data set. Scales show patristic distances while circle sizes represent RAxML bootstrap support of each division.

Mean patristic distance (i.e., branch length) was significantly less in intraindividual than in unrelated comparisons in phylogenies of *B. adolescentis*, B. longum, and *B. bifidum* strains (bootstrapped Wilcoxon rank sum *P* < 0.05 in all three cases, Fig. 3). This result indicates strain stability: an individual’s strains are more similar for the same person sampled over time compared to strains from a different person. We did observe some instances of large patristic distance between samples of the same individual (Fig. 4), and in both cases where three samples per individual were included, one of the three samples had a large patristic distance more typical of unrelated comparisons (Fig. 4). These large interindividual patristic distances could result from detection errors (i.e., a strain was present in both samples but failed to be detected in one), or from differences in the relative abundance of strains between time points (this analysis picks the most abundant), or from loss and regain of strains.

Mean patristic distance of same-family comparisons was not significantly different from that of unrelated comparisons for any of the four taxa examined (Wilcoxon rank sum of mean distances in intertwin versus unrelated, *P* > 0.05 in all cases, Fig. 3). Thus, the MLSA-based RAxML phylogenies did not show that co-twins harbored similar strains of these 4 species. Furthermore, this analysis did not reveal any significant grouping of strains according to lactase genotype (ANOSIM permutations of phylogenetic distance matrix *P* > 0.05, Fig. 4) and longitudinal intraindividual comparisons were not significantly different between genotype groups for any of the four species we examined either individually (Fig. S2) or in aggregate (Fig. S3) (Wilcoxon rank sum test of mean intraindividual distance, AA versus GG, *P* > 0.05; Table 2A and Table S1, tab 5). The time interval between repeated samples from the same individual and patristic distance did not reveal any relationship for any species (Spearman’s rho nonsignificant in all cases, Fig. S4), supporting strain stability with time.

**TABLE 2:** Overview of analyses for each of the four species examined at the strain level and for all species combined in aggregate^*a*^

			Adol	Long	Bifi	Anim	In aggregate
A	Marker gene-based phylogeny (StrainPhlAn)	Longitudinal stability	Yes	Yes	Yes	No	Yes
		Stability GG > stability AA/AG	No	No	NA	NA	No
		Twin effect	No	No	No	No	No
B	% of shared marker-SNPs (MIDAS)	Longitudinal stability	Yes	Yes	Yes	No	Yes
		Stability GG > stability AA/AG	Yes	Yes	NA	NA	Yes
		Twin effect	Yes	No	No	No	No
C	Synteny (all regions for each species in aggregate)	No. of gene regions	7	10	4	0	21
		Longitudinal stability	6/7	4/10	2/4	NA	Yes
		Stability GG > stability AA/AG	0/2	0/8	0/0	NA	No
		Twin effect	4/7	0/8	1/4	NA	No
D	Enriched in GG vs AA/AG (16S rRNA SVs)		Yes	Yes	Yes	No	Yes

a Panels A to C show significance for our three strain-level approaches. StrainPhlAn results are based on best tree values from the RAxML hill-climbing algorithm. Within each panel, “longitudinal stability” refers to significantly greater similarity of Intra (same person over time) versus Unrelated (between unrelated people) comparison categories. “Stability GG > stability AA/AG” refers to greater mean “Intra” values for GG individuals than for AA/AG individuals (i.e., is there greater stability within lactase-nonpersistent than lactase-persistent individuals). Finally, “Twin effect” refers to greater similarity of Same Family (between twin siblings) than Unrelated comparison categories. Panel D shows significance of genotypic enrichment from 16S rRNA SV data across the broad TwinsUK data set (*n* = 1,680). In each panel, “yes” and “no” refer to significance of the statistical test as described in the main text, while “NA” indicates that 3 or fewer samples were available in at least one category. In panel C (synteny), the number of significant regions out of the total number of regions with sufficient comparisons is shown. *P* values are shown in Table S1, tabs 5 and 6. Species abbreviations are as follows: Adol, *B. adolescentis*; Long, B. longum; Bifi, *B. bifidum*; Anim, *B. animalis*.

10.1128/mSystems.00911-20.2 FIG S2 Host genotype effects on intraindividual longitudinal strain stability. All intraindividual longitudinal comparisons for each of B. longum, *B. adolescentis*, and *B. bifidum* are plotted according to host genotype for patristic distance (branch lengths) between samples across our RAxML strain phylogenies (A) and the percent marker-SNPs shared between samples (B). Significance levels are Wilcoxon rank sum tests. ns, *P* > 0.05; *, *P* ≤ 0.05; **, *P* ≤ 0.01; ***, *P* ≤ 0.001; ****, *P* ≤ 0.0001. Red diamonds represent mean values. Download FIG S2, PDF file, 0.1 MB. Copyright © 2020 Schmidt et al. 2020 Schmidt et al. This content is distributed under the terms of the Creative Commons Attribution 4.0 International license.

10.1128/mSystems.00911-20.3 FIG S3 Host genotype effects on longitudinal strain stability and strain similarities between monozygotic twins across all four species in aggregate. All three metrics of strain comparisons are shown, with each separated by lactase persistence genotype and the category of comparison: Intra (same person over time), same family (comparisons between twin siblings), and unrelated (comparisons between unrelated people). Plots represent combined data for B. longum, *B. bifidum*, and *B. adolescentis.* The metrics shown for each comparison category are patristic distance (branch lengths) between samples across our RAxML strain phylogenies (A), the percent marker-SNPs shared (B), and synteny scores across the phylogenies (C). Significance levels are median Wilcoxon rank sum tests after 999 bootstraps to smallest group size. ns, *P* > 0.05; *, *P* ≤ 0.05; **, *P* ≤ 0.01; ***, *P* ≤ 0.001; ****, *P* ≤ 0.0001. Download FIG S3, PDF file, 0.2 MB. Copyright © 2020 Schmidt et al. 2020 Schmidt et al. This content is distributed under the terms of the Creative Commons Attribution 4.0 International license.

10.1128/mSystems.00911-20.4 FIG S4 Temporal distance between intraindividual samples is not correlated with patristic distance across MLSAs. All unique intrapersonal comparisons are plotted, with time between samples on the *x* axis and StrainPhlAn MLSA patristic distance on the *y* axis. Blue line shows least-squares regression with 95% confidence intervals, while Spearman’s rho and the significance of the correlation are shown. Download FIG S4, PDF file, 0.1 MB. Copyright © 2020 Schmidt et al. 2020 Schmidt et al. This content is distributed under the terms of the Creative Commons Attribution 4.0 International license.

### Marker-SNPs are shared at higher percentages in intraindividual than in unrelated comparisons.

**FIG 5:**
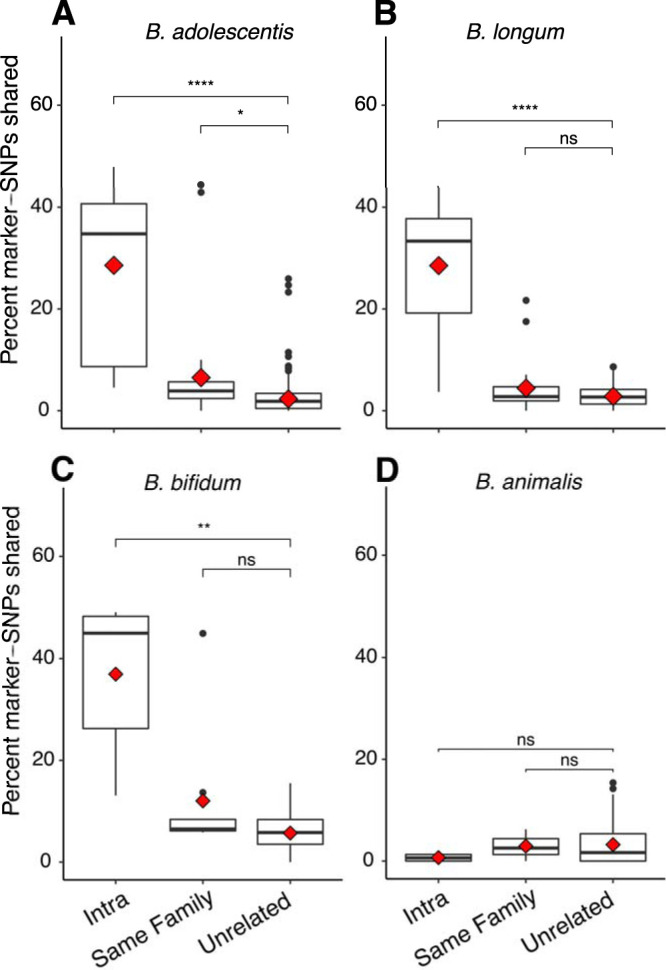
Marker-SNP sharing over time and between individuals shows strain stability and twin sharing in three of four species. Percentage of marker-SNPs shared for all pairwise comparisons, separated by the category of comparison: intra (same person over time), same family (comparisons between twin siblings), and unrelated (comparisons between unrelated people). Shown are the four taxa for which sufficient coverage existed to calculate marker-SNPs. Species are *B. adolescentis* (A), B. longum (B), *B. bifidum* (C), and *B. animalis* (D). Boxplots show median and quartiles, while red diamonds show means. Significance levels are median Wilcoxon rank sum tests after 999 bootstraps to smallest group size. ns, *P* > 0.05; *, *P* ≤ 0.05; **, *P* ≤ 0.01; ***, *P* ≤ 0.001; ****, *P* ≤ 0.0001.

To further interrogate *Bifidobacterium* strain stability within individuals over time, we employed a “strain tracking” feature, implemented in the MIDAS software, to identify rare marker-SNPs highly discriminant for a specific strain in an individual at a single time point. We had sufficient coverage to employ this approach for each of B. longum, *B. adolescentis*, *B. animalis*, and *B. bifidum* species (Table 1). Our results show that microbe marker-SNPs of an individual are far more likely to be found in the same individual at a different time point than in an unrelated individual in three of the four species examined (bootstrapped Wilcoxon rank sum of mean percent shared SNPs, intraindividual versus unrelated, *P* < 0.0001 in all cases, Fig. 5). *B. animalis* was the one exception, where SNP sharing was not significantly higher in intraindividual than in unrelated comparisons (bootstrapped Wilcoxon rank sum *P* = 0.32; Fig. 5, Table 2B, and Table S1, tab 5).

We observed greater mean intraindividual sharing of *B. adolescentis* and B. longum in GG than in AA individuals (Fig. S2), as well as when all species were considered in aggregate (Wilcoxon rank sum of mean intraindividual percent shared SNPs, GG versus AA individuals, *P* = 3.3e^−9^; Fig. S3). This pattern was driven by B. longum and *B. adolescentis*, the two most abundant species (Fig. S2).

**FIG 6:**
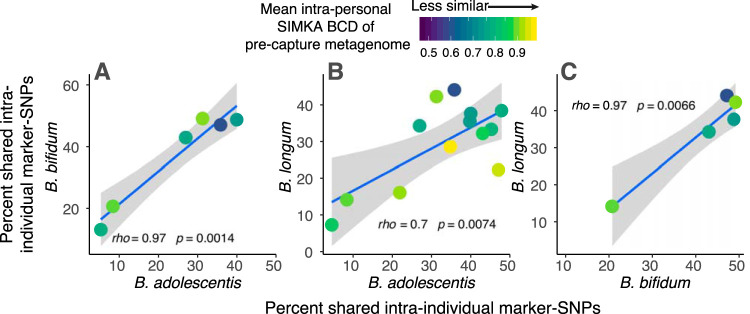
Intraindividual marker-SNP sharing across different *Bifidobacterium* species. Each plot shows the percentage of marker-SNPs shared within an individual over time for two different *Bifidobacterium* species; each of three comparison possibilities is shown across plots A, B, and C. Each point represents a comparison between longitudinal samples from the same individual. Points are colored by the Simka Bray-Curtis Dissimilarity (BCD) value between the two samples, as measured from precapture metagenomes, and is therefore a measure of overall microbiome dissimilarity over time within an individual. Blue line shows least-squares regression with 95% confidence intervals, and Spearman’s rho along with the significance of the correlation is shown.

The marker-SNP analysis revealed that high stability in either B. longum, *B. bifidum*, or *B. adolescentis* was a good predictor of stability in the other two species within that same individual (Fig. 6). This suggests some individuals carry stable strains, while others witness strain replacement, across all three species together. We detected higher marker-SNP sharing within families (i.e., a twin effect) for *B. adolescentis* (bootstrapped Wilcoxon rank sum *P* = 0.021) but not the other three species examined, possibly due to a lower power to detect this pattern in *B. bifidum* (for which synteny analysis shows a twin effect [see below]). The percentage of shared marker-SNPs for a given intraindividual comparison was not correlated with time between sample collection, nor with other metrics of overall microbiome similarity (Fig. S5), meaning the percentage of shared marker-SNPs between samples does not decrease with time, and the patterns observed here are therefore not a function of time-induced SNP accumulation.

10.1128/mSystems.00911-20.5 FIG S5 Temporal distance between intraindividual samples is not correlated with overall microbiome community similarity, synteny, or mean marker-SNP sharing. All unique intrapersonal comparisons are plotted, with time between samples on the *x* axis and a metric of stability of similarity on the *y* axis. The metrics are as follows: Bray-Curtis Dissimilarity (BCD) based on SIMKA k-mer spectra of precapture metagenomes (A), BCDs based on metabolic pathways of precapture metagenomes (B), mean synteny scores across all regions examined (C), and mean percentage of marker-SNPs shared across all four taxa (D). Blue line shows least-squares regression with 95% confidence intervals, while Spearman’s rho and the significance of the correlation are shown. Download FIG S5, PDF file, 0.1 MB. Copyright © 2020 Schmidt et al. 2020 Schmidt et al. This content is distributed under the terms of the Creative Commons Attribution 4.0 International license.

Notably, *Bifidobacterium* strains exhibited intraindividual longitudinal stability despite high variability in overall microbiome communities around them. Only a very weak, nonsignificant association was observed between an individual’s overall microbiome stability (assessed using Bray-Curtis Dissimilarity [BCD] of precapture metagenomes) and the number of shared marker-SNPs for a given *Bifidobacterium* strain (Fig. 6 and Fig. S6). This pattern held when microbiome community dissimilarity was assessed using either Simka BCD values, a k-mer-based annotation-independent method, or BCD values for annotations of metabolic pathways of precapture metagenomes (Fig. S6). These results suggest *Bifidobacterium* stability may be independent from the dynamics of microbiome communities in which the bacteria exist.

10.1128/mSystems.00911-20.6 FIG S6 Intraindividual marker-SNP sharing of *Bifidobacterium* species does not correlate with overall community dissimilarity. Percentage of *Bifidobacterium* marker-SNPs shared between samples from the same individual over time, plotted against the overall community dissimilarity from those samples, based on precapture metagenomes (i.e., standard metagenome libraries). BCD metrics used to assess overall community dissimilarity are Simka, a k-mer-based dissimilarity metric between the two samples (A), and BCD values for MetaCyc annotations of metabolic pathways generated in HUMAnN2 (B). Blue line shows least-squares regression with 95% confidence intervals, and Spearman’s rho along with the significance of the correlation is shown. Download FIG S6, PDF file, 0.1 MB. Copyright © 2020 Schmidt et al. 2020 Schmidt et al. This content is distributed under the terms of the Creative Commons Attribution 4.0 International license.

### Synteny analysis reveals sharing of strains within twin pairs and strain persistence in individuals over time.

The analyses described above are based on single nucleotide variations and gene-content comparisons. Gene synteny is defined as the conservation of gene order between chromosomes, along an evolutionary gradient (17), and was previously used as a measure of the distance between genomes (18, 19). Here, we identify syntenic blocks, regions of conserved DNA sequence occurring in the same order in two different chromosomes, to determine the relatedness of *Bifidobacterium* strains. We created a pipeline to compare the synteny of different genomic regions in three *Bifidobacterium* species for which we had sufficient coverage from captured metagenomes: B. longum, *B. adolescentis*, and *B. bifidum* (see Materials and Methods). Briefly, we performed a *de novo* assembly of postcapture metagenomes in each sample and used a subset of randomly selected genes to perform a BLAST search against the metagenomic assemblies. Next, we performed a pairwise synteny comparison between all genomic regions, which included the gene used as BLAST query and its flanking regions (3.5 kbp upstream and downstream of the gene). Finally, we defined a synteny score and performed the same pairwise comparisons as outlined above for regions which were identified in >15 single-individual assemblies. In summary, we analyzed 10, 7, and 4 regions for B. longum, *B. adolescentis*, and *B. bifidum*, respectively.

**FIG 7:**
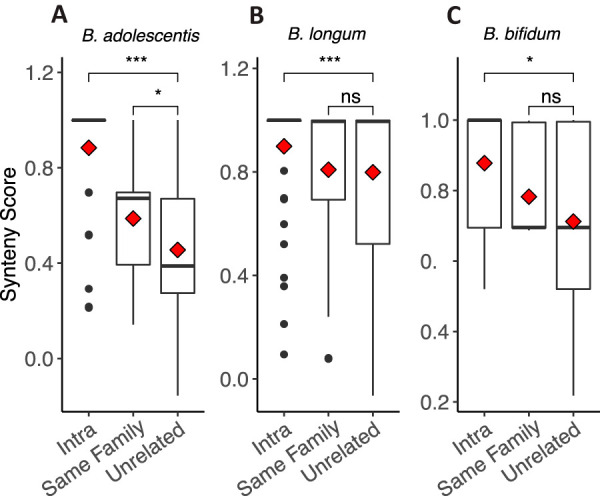
Within-species synteny scores over time and between individuals. Pairwise synteny scores for genomic regions of the three *Bifidobacterium* species for which sufficient read depth was generated for synteny analyses. Plots show all pairwise comparisons, separated by the category of comparison: intra (same person over time), same family (comparisons between twin siblings), and unrelated (comparisons between unrelated people). Species are *B. adolescentis* (A), B. longum (B), and *B. bifidum* (C). Boxplots show median and quartiles, while red diamond shows mean. For each species, all regions were combined into a single analysis. Significance levels are Wilcoxon rank sum tests. ns, *P* > 0.05; *, *P* ≤ 0.05; **, *P* ≤ 0.01; ***, *P* ≤ 0.001; ****, *P* ≤ 0.0001.

For all three species, intraindividual comparisons yielded significantly higher synteny scores than comparisons of unrelated individuals (Fig. 7), although this pattern varied somewhat by region; 6/7 genomic regions for *B. adolescentis*, 2/4 regions for *B. bifidum*, and 4/10 regions for B. longum (results are summarized in Table 2C and *P v*alues are shown in Table S1, tab 6). These results indicate that the same strains persist in the gut over time. Comparisons of synteny between individuals within a twin pair yielded significantly higher scores (relative to comparisons of unrelated individuals) in 4 regions in *B. adolescentis* and in one region in *B. bifidum*. In B. longum, although not statistically significant, 3 regions showed lower synteny scores for individuals within a pair (*P* values 0.055 to 0.08, Table S1, tab 6). Finally, *LCT* genotype did not influence longitudinal strain stability as measured by synteny. No differences were seen in mean intraindividual synteny scores between lactase persistence genotypes (Wilcoxon rank sum test of mean intraindividual synteny, AA versus GG, *P* > 0.05), and each genotype independently showed significant differences between intraindividual and unrelated comparisons (bootstrapped Wilcoxon rank sum test, *P* = 0.0005 for AAs and *P* = 6.1e^−5^ for GGs, Fig. S3). Together, these results support strain-sharing events within the families and maintenance of strains over time within individuals.

## DISCUSSION

One of the strongest signals of host genetic effects on microbiome communities is the association between *Bifidobacterium* and an SNP in the regulatory region of the lactase gene *LCT*. Several independent studies have noted a greater relative abundance of the genus *Bifidobacterium* in the gut microbiomes of lactase-nonpersistent compared to lactase-persistent individuals of European origin (5, 7–9). We did not detect an interaction between dairy consumption, *LCT* genotype, and levels of *Bifidobacterium* here, as reported by Bonder et al. (9). However, our results show that compared to the lactase-persistent genotypes (AA/AG), the lactase nonpersistence genotype (GG) enhances the proportion of prevalent and abundant *Bifidobacterium* species, without bias toward particular species, strains, or genome content. Our results also indicate that strains of certain *Bifidobacterium* species are shared within twin pairs and persist over time within individuals against a background of a dynamic microbiome community.

Corroborating results of previous reports (20–22), we observed that *B. adolescentis* and B. longum dominated the *Bifidobacterium* communities of the adult gut microbiome. Strain comparisons within an individual over time indicated that *B. adolescentis*, B. longum, and *B. bifidum* were stable within adults over multiyear timescales as reveled by multiple methods. Strain sharing between twins showed a weaker signal in general, with *B. adolescentis* as the only species with a significant twin effect across multiple methods. Mechanisms leading these four species to vary in regard to longitudinal stability or twin effects remain to be elucidated. Nevertheless, it is interesting that *B. animalis*, the one species for which stability was not shown, was also among the few species not enriched in GG versus AA/AG genotype groups from our broader 16S rRNA and metagenomic surveys. These observations may reflect a different ecology of *B. animalis* compared to the others. *B. animalis* is commonly isolated from dairy and other sources outside the human microbiome (23) and has a reduced genetic repertoire for carbohydrate metabolism versus other taxa in the genus (24). *B. animalis* may therefore be a more transient autochthonous member of the adult gut microbiome. It is also important to consider that *B. adolescentis* and B. longum both showed the highest number of significant patterns across our methods and had the largest sample numbers. The lack of significant findings for *B. animalis* and *B. bifidum* must therefore be considered in light of the low power attributable to their analyses. Adding additional samples with future research could confirm that variability in longitudinal stability between species and strains is not due to sample number biases.

A leading hypothesis for the greater relative abundance of *Bifidobacterium* in lactase-nonpersistent individuals is a greater availability of undigested lactose in the large intestine, which may be preferentially used by bifidobacteria (9, 25). The availability of undigested lactose in the guts of lactase-nonpersistent individuals could in principle result in niche partitioning between the different species. Indeed, many taxa within the *Bifidobacterium* genus are considered specialists (20, 26), and some devote significant proportions of their genomes to particular host-derived resources including human milk oligosaccharides in Bifidobacterium longum subsp. *infantis* (27), and mucin glycans in *B. bifidum* (26). B. longum subsp. *infantis*, for example, is known to specialize in human milk oligosaccharide metabolism, while Bifidobacterium longum subsp. *longum* is specialized in metabolism of plant-derived carbohydrates (10, 28). Furthermore, gut *Bifidobacterium* taxa are known to follow successional patterns with a gradual shift from B. longum subsp. *infantis*, *B. breve*, and *B. bifidum* in infant guts to B. catenulatum, *B. adolescentis*, and B. longum subsp. *longum* in adults (21, 22). These shifts are thought to result in part from shifts in diet and are associated with breastfeeding versus formula, weaning, and intake of solid food (29, 30). However, our results instead point to lactose utilization as enhancing all the common *Bifidobacterium* species, without altering their population structure. This implies that in the adult gut microbiome, common *Bifidobacterium* species use lactose without competing for it and it does not become a limiting resource. The nondifferentiating effect of host *LCT* genotype on the *Bifidobacterium* community results suggests a “rising tide raises all boats” scenario, where a lactose utilization advantage is equally distributed among common *Bifidobacterium* species.

The strain comparisons in this study relied on enrichment of *Bifidobacterium* DNA by genome capture. This worked well for the most abundant species, *B. adolescentis* and B. longum, and allowed a reduced set of comparisons possible for *B. bifidum* and *B. animalis* due to lower coverage. To compare strains of the other bifidobacteria present would require deeper sequencing or a more tailored set of probes in the genome capture. To compare strains, we used two published methods based on sequence composition: one using an alignment of marker-genes (StrainPhlAn) and another using individually discriminant marker-SNPs (MIDAS). The novel synteny-based method that we developed allows for the identification of differences in the organization of genomic regions even when the sequence similarity is high. Generally, the two published methods agreed with the synteny approach, with some discrepancies (Table 2). All three methods detected intraindividual longitudinal stability for *B. adolescentis*, *B. bifidum*, and B. longum, while no method detected stability in *B. animalis.* The three methods were divided, however, in their detection of within-family sharing of strains (a twin effect) and on the influence of lactase persistence genotype on strain stability. Both the marker-SNP and synteny approaches detected greater similarity of *B. adolescentis* strains between twins than between unrelated individuals, while our marker gene alignments did not. Only the synteny approach detected twin sharing in B. longum and *B. bifidum*. The marker-SNP approach was the only method to reveal any influence of lactase persistence on longitudinal stability and did so only in *B. adolescentis* and B. longum. Why each method varied in its ability to detect a particular pattern is not clear; however, their agreement with regard to longitudinal stability of *B. adolescentis*, B. longum, and *B. bifidum* gives strong support to the conclusion that these species are stable within adults over multiyear timescales.

Temporal stability, along with vertical transmission within families, is expected to be associated with heritability. In addition to temporal stability and within-family sharing of strains shown here, we had previously noted that the genus *Bifidobacterium* was both heritable and stable over time, based on 16S rRNA gene analysis (31). For the relative abundances of microbial taxa or genes to be stably heritable over time, they must be present every generation and associated with host genetic variation. If the acquisition of microbes from the environment or other individuals is less reliable than acquisition from parents, the heritability of horizontally acquired microbes should be less stable over time than the heritability of vertically acquired microbes. These results, together with previous observations, continue to reveal the bifidobacteria as highly human-adapted members of the microbiome.

*B. adolescentis*, B. longum, and *B. bifidum* have also previously been shown to be temporally stable in the gut microbiomes of infants, where they are far more abundant than in the adult gut microbiome. Strains of these species were stable within children for up to 3 years of age across diverse geographic cohorts (30), and B. longum subsp. *longum* was stable from infancy until 6 years of age (32). Furthermore, *B. bifidum* and B. longum are transmitted from mother to infant (33, 34). By extending existing evidence of *Bifidobacterium* strain stability to an adult cohort and demonstrating strain sharing within families for *B. adolescentis*, our results contribute to an overall picture of this genus, one which suggests long-term maintenance of specific strains within an individual which can be transmitted across generations.

## MATERIALS AND METHODS

### Sample inclusion and access.

All existing samples from the TwinsUK cohort were included assuming they had both (i) metagenomic or 16S rRNA sequence data and (ii) genotype data at the 13910 G/A allele. All work involving the use of these previously collected samples was approved by the Cornell University IRB (protocol ID 1108002388).

### 16S rRNA gene-based community analyses.

16S rRNA reads were extracted from existing data sets (*n* = 1,680) and analyzed via the Qiime2 pipeline (35) with minor deviations. Briefly, PCR amplicons for the V4 region of the 16S rRNA gene were generated with primers 515F-806R and were sequenced with the Illumina MiSeq 2 × 250 v2 kit at the Cornell University Institute for Biotechnology as previously described (5). DADA2 (36) was used to call 100% sequence identity sequence variants (SVs, also known as 100% OTUs or ASVs). Taxonomy was assigned to SVs with the QIIME2 q2-feature-classifier (37) using the SILVA database (v119) (38). Taxonomic annotations of 16S rRNA sequence variants (SVs) were improved from the standard DADA2 output by extracting each SV representative sequence and querying it against the NCBI type strain database. In one case all NCBI annotations fell within the B. longum*/B. breve* clade, and it was thus assigned as “B. longum*/breve*” in subsequent analyses; in all other cases the original DADA2 assignment was kept.

Statistical testing for the influence of genotype was done using a nonparametric Wilcoxon rank sum test of the total relative abundance of all *Bifidobacterium* SVs between genotypes. Only SVs that occurred in at least 2 individuals across the data set of 1,680 samples were included. Individuals with either AA or AG at 13910*A (rs4988235) were considered lactase persistent, while only GG was considered lactase nonpersistent. Independent Wilcoxon rank sum tests were run for each *Bifidobacterium* SV independently across the two genotype groups. Because our data set included replicate samples from the same individual (therefore not independent), we determined the overall influence of genotype on *Bifidobacterium* SV relative abundance using a linear mixed-effect model, with participant ID as a random effect, in the R package “lmr4”: *Bifidobacterium*RA ∼ Genotype + (1 | ParticipantID).

To assess the community structure of the genus without the influence of an overall enrichment in GG individuals, all SVs annotated as “bifidobacterium” were extracted into new SV tables and again normalized by 1 within an individual and then input into LEfSe (12) via the Galaxy web interface with default parameters, genotype set as class, and no subclass. Longitudinal analyses of *Bifidobacterium* SVs narrowed the total sample count to 556 (278 matched pairs), and Spearman’s rho was calculated assigning time 1 and time 2 to one sample or another at random. To overcome unequal sample sizes (GG = 30, AA/AG = 248), a mean Spearman rho was calculated after subsampling to *n* = 15 for each genotype over 999 permutations. All analyses were done in RStudio (v. 1.0.136).

Dairy intake was assessed as the portion size frequency for dairy for a week, adjusted for energy intake, using food frequency questionnaires developed and described elsewhere (50). Associations with *Bifidobacterium* SVs were conducted using linear mixed-effect models, with genotype as a random effect, in the R package “lmr4”: *Bifidobacterium*RA ∼ DairyFrequency + (1 | ParticipantID). Statistical significance was assigned using Satterthwaite’s method in the R package “lmerTest” (39).

### Metagenomic community analyses.

Metagenome sequences were used from existing data sets (*n* = 245) which were extracted as described above with library preparation previously described (40). Taxonomic assignments were made using Kraken2 (41). We assessed the influence of genotype on the relative abundance of *Bifidobacterium* annotations using a mixed-effect model as described above. Metabolic pathway annotations were generated using the HUMAnN2 pipeline against the MetaCyc database (42), and significantly discriminant gene pathways were revealed using HUMAnN2’s built-in ‘humann2_associate’ script with default parameters at the gene pathway level (42).

### Subset for genome capture, strain-level analyses, and discriminant functional pathways.

An additional subset of the TwinsUK cohort was selected for genome capture based on (i) the availability of genotype data at the lactase persistence gene (rs4988235) and (ii) at least 2 longitudinal samples between 8 months and 4 years apart with a body mass index (BMI) change of less than 3. We note that variation in temporal distance between sampling events had no impact on stability, community composition, or synteny values (see Fig. S4 and S5 in the supplemental material). In total 20 individuals with 2 time points and 2 individuals with 3 time points were selected (a total of 46 samples across 11 GG individuals/11 AA individuals). Each genotype also contained 4 sets of twin siblings (Table S1, tab 2). DNA samples were brought through metagenome creation, capture reaction, and sequencing according to NimbleGen (Madison, WI, USA) SeqCap EZ HyperCap Workflow v.1.0. Briefly, genomic DNA (gDNA) underwent enzymatic fragmentation and adapter-mediated PCR using KAPA HyperPlus library preparation, followed by a 16-h hybridization with a custom set of biotinylated long oligonucleotide probes (the “probe array”), followed by a final reamplification. The probe array was designed and manufactured by NimbleGen and included overlapping coverage of a total capture space of >94 Mb and 89k capture targets which covered 47 type strain *Bifidobacterium* genomes (Table S1, tab 3). Precapture and postcapture metagenomes were then sequenced across two Illumina paired-end 300-cycle sequencers (HiSeq 3000).

Analyses on each pre- and postcapture library from our 46-sample longitudinal subset (92 metagenomes total) were conducted with Simka, an annotation-independent k-mer-based metric (43), and the annotation-based HUMAnN2 pipeline against the MetaCyc database (42). Simka Bray-Curtis Dissimilarity (BCD) matrices were calculated directly within the software. HUMAnN2 BCD matrices were generated from gene pathway relative abundance tables, after removal of collapsed pathway stratifications, using vegdist in the Vegan R package (V. 1.0.136). Each pairwise comparison within the BCD matrices was then classified into one of three categories: intraindividual (same person over time), same family (comparisons between twin siblings), and unrelated (comparisons between unrelated people). Wilcoxon rank sum tests were used to determine differences between comparison categories. To overcome issues of nonindependence related to multiple samples from the same individual within the data set, we ran 9,999 permutations of Wilcoxon rank sum tests using subsets of individuals equal to the smallest number of samples from either category and reported the mean value. Finally, ANOSIM tests of BCD hierarchical clustering were conducted using the ‘anosim’ script part of the ‘vegan’ R package with 9,999 permutations. ANOSIM permutation tests were run on intraindividual clusters, unrelated clusters, and same-family clusters.

### MIDAS marker-SNPs and StrainPhlAn MLSAs.

Strain-level assessments were made using the Metagenomic Intra-Species Diversity Analysis System (MIDAS) (16) and StrainPhlAn (15). MIDAS’s ‘strain_straintracker.py’ program was used with default settings to identify marker-SNPs as previously described (16). Briefly, MIDAS initially identifies abundant species by mapping unassembled metagenomic reads to a database of 30,000 reference genomes and then identifies per-species SNPs for each abundant species within a sample. To identify the SNPs used in our analyses, we had MIDAS identify rare, sample-discriminatory SNPs from a pool of 1 sample per individual (i.e., SNPs that were unique to that individual). We term these sample-discriminatory SNPs “marker-SNPs.” Each individual’s additional sample(s) was then added back into the pool, and marker-SNP overlap was assessed between sets of two samples in all possible pairwise comparisons across the data set. The percentage of shared marker-SNPs was the number of marker-SNPs shared between two samples, divided by the total number across both individuals. Bootstrapped Wilcoxon rank sum tests were performed on the mean percentage of marker-SNPs shared in each comparison category described above (i.e., intraindividual, same family, and unrelated). Bootstrapping was done down to the lowest number in a single category for any given comparison and run for 9,999 permutations, and the mean *P* value was reported.

StrainPhlAn multilocus sequence alignment (MLSA) phylogenies were created using the standard RAxML hill-climbing algorithm, as described elsewhere (15, 33). Bootstrap values were generated using the rapid bootstrapping RAxML algorithm with 100 iterations (44). Resulting phylogenies were uploaded into the ITOL program (45) for graphical display of family and twin ID annotations, and best tree phylogenies from the hill-climbing algorithm were uploaded into Geneious (v 6.1.8) to retrieve patristic distances (branch lengths). Mean patristic distances were compared across comparison categories using bootstrapped Wilcoxon rank sum tests as described above.

All software was run parallelized on a high-performance computing cluster at the Max Planck Institute for Developmental Biology via Snakemake (46).

### Synteny analyses.

First, a *de novo* assembly of postcapture metagenomes was performed for each sample by using the MetaCompass software (47). Next, a set of genes representing different genomic regions was selected randomly (using a custom script) from each of the reference genomes of B. longum, *B. adolescentis*, *B. animalis*, and *B. bifidum.* Each gene was used as a query for a BLASTn (48) search against the assembled metagenomes, with minimal identity of 97% and minimal coverage of 90%. For each of the blast hits in the assembled metagenomes, the gene and flanking sequences (3.5 kb upstream and downstream of the blast hit) were retrieved and used for synteny comparison. Further analysis was carried out only for regions found in >15 samples. If for a given species the final number of suitable regions was lower than 4, the process iterated with a new set of genes, keeping a minimal gap of 5 genes between each two selected genes, to avoid overlap between regions.

Pairwise synteny comparison between each two DNA sequences was done using the DECIPHER R package (49). Breaks in the synteny were defined as nonhomologous regions longer than 15 bp. To compare the synteny between different types of pairwise comparisons (i.e., intraindividual, twins, and nonrelated individuals) we defined the synteny score (equation 1):

(1) $$\mathrm{Syn}\_\mathrm{score}=1+\log_{10}\frac{\sum_{1}^{n}\mathrm{Lsb}(n)}{\mathrm{Lseq}\times n}$$

where *n* is the number of synteny blocks identified in each pairwise comparison, *Lseq* is the length of the shorter sequence in each pair of compared sequences, and *Lsb(n)* represents the length of the *n*th synteny block.

We performed a validation of this synteny method using a set of 15 Escherichia coli genomes classified to two different clades, either K-12 MG1655 or O157:H7 (Table S1, tab 7A). We started our analysis by randomly selecting five genes from the E. coli O157:H7 reference genome (NCBI reference sequence NC_002695.2) and analyzing the genomic regions flanking each target gene (10 kb up- and downstream of the gene). Two genes (*mnmC* and *ubiD*) were found in high homology only in O157:H7 genomes while the other genes (*polB*, *hemA*, and *nupG*) were detected in genomes of both groups. Pairwise comparisons of all three regions showed with high significance that the within-clade synteny scores are higher than the between-clade synteny scores (Table S1, tab 7B, and Fig. S7).

10.1128/mSystems.00911-20.7 FIG S7 Synteny method validation with E. coli genome. Synteny scores of pairwise comparisons, for 3 regions in the E. coli genome (see Materials and Methods). Pairwise comparisons are grouped by the type of the pair, either within clade or between clades. Panel titles denote the gene located at the center of each genomic region. Download FIG S7, PDF file, 0.1 MB. Copyright © 2020 Schmidt et al. 2020 Schmidt et al. This content is distributed under the terms of the Creative Commons Attribution 4.0 International license.

### Ethics approval and consent to participate.

All work involving the use of these previously collected samples from human subjects was approved by the Cornell University IRB (protocol ID 1108002388).

### Availability of data and materials.

Sequences of pre- and postcapture metagenomes (the only new data generated for this paper) are available on the European Nucleotide Archive under accession number PRJEB38000.
