# Supplementary figures and images for: Strain-Level Analysis of Bifidobacterium spp. from Gut Microbiomes of Adults with Differing Lactase Persistence Genotypes
Source: mSystems. 2020 Sep 29;5(5):e00911-20. doi: 10.1128/mSystems.00911-20 (PMC7527142; doi:10.1128/mSystems.00911-20)

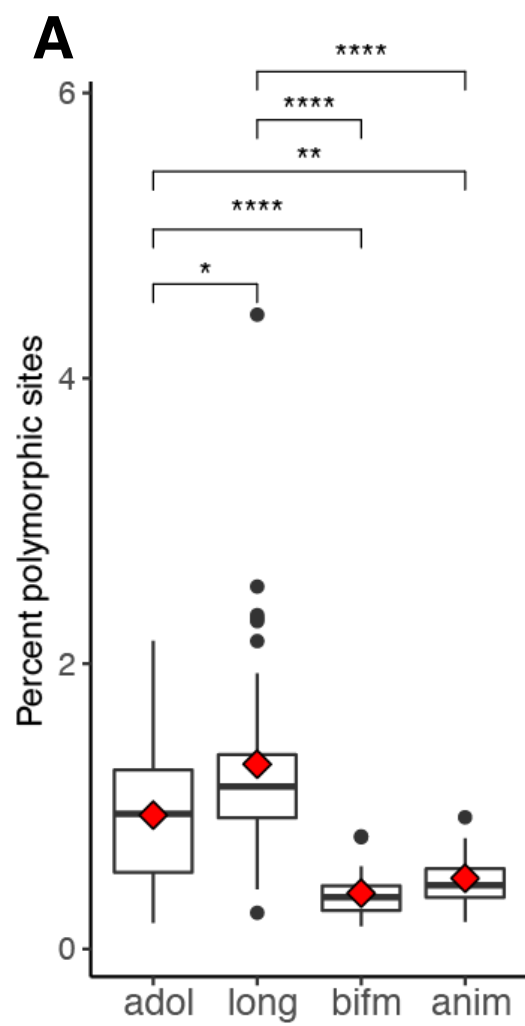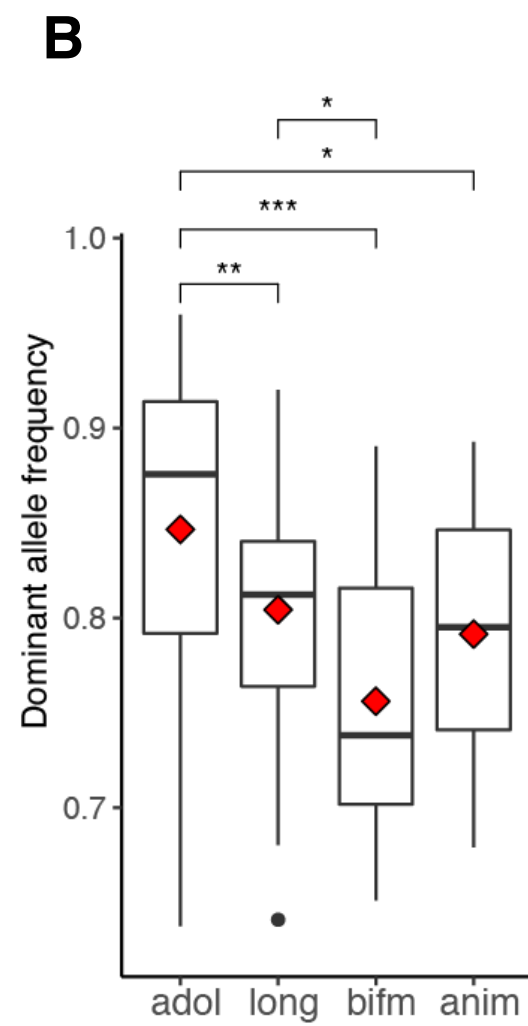

Supplement: FIG S1 [file mSystems.00911-20-sf001.pdf]

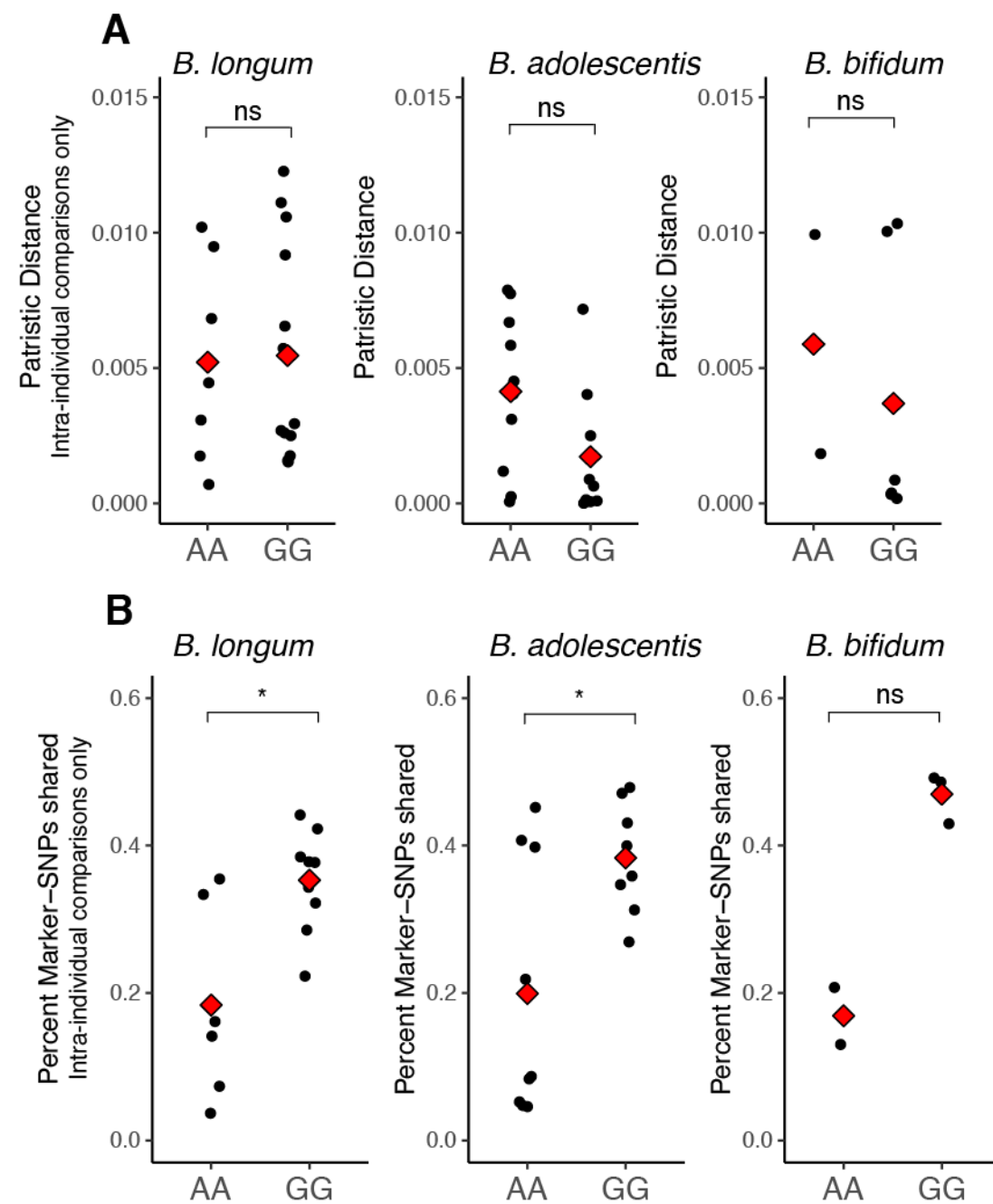

Supplement: FIG S2 [file mSystems.00911-20-sf002.pdf]

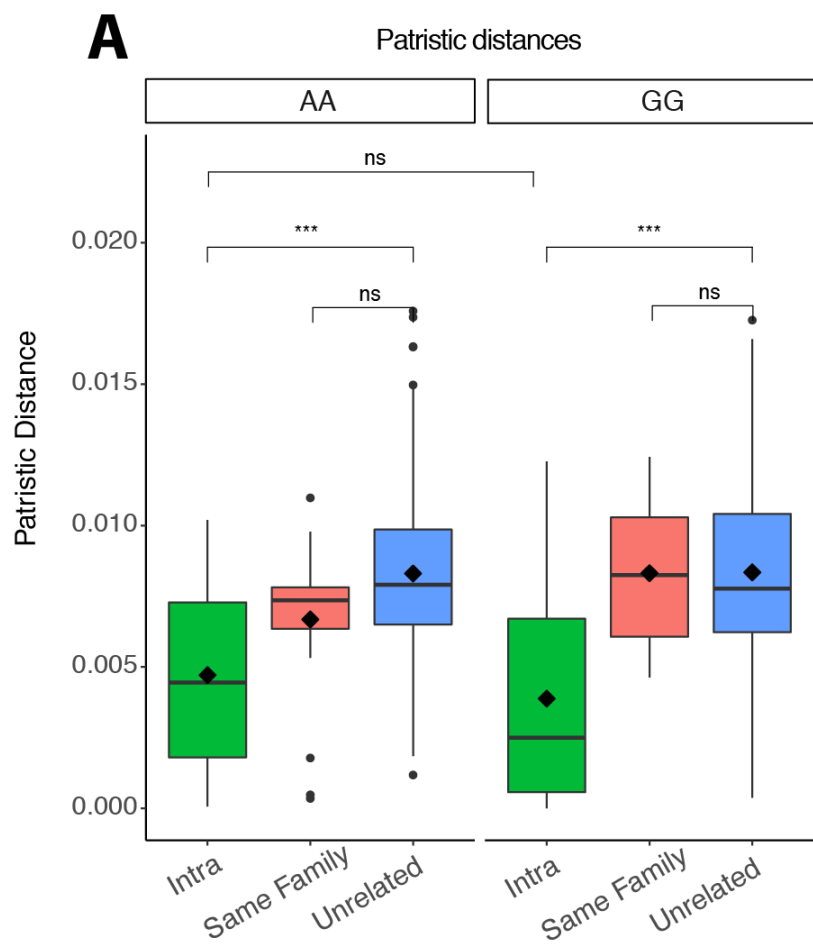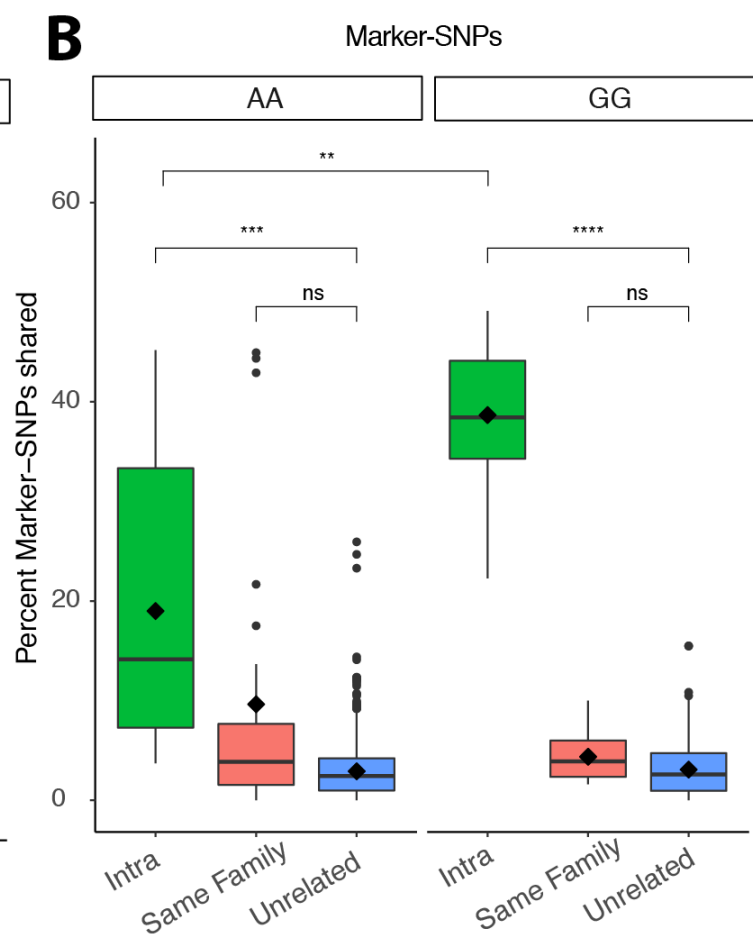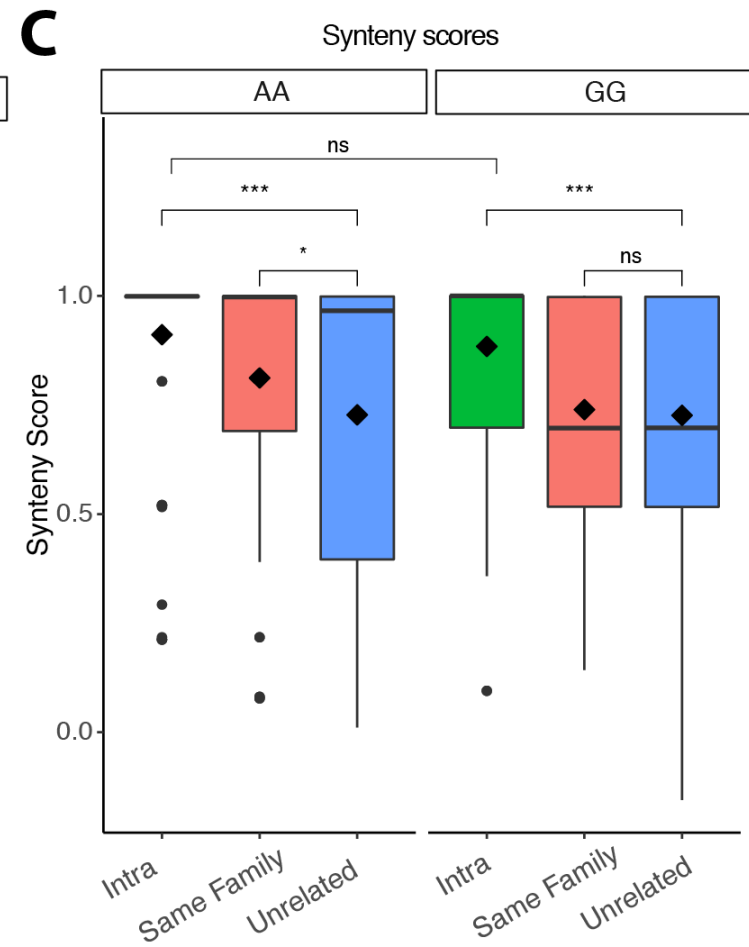

Supplement: FIG S3 [file mSystems.00911-20-sf003.pdf]

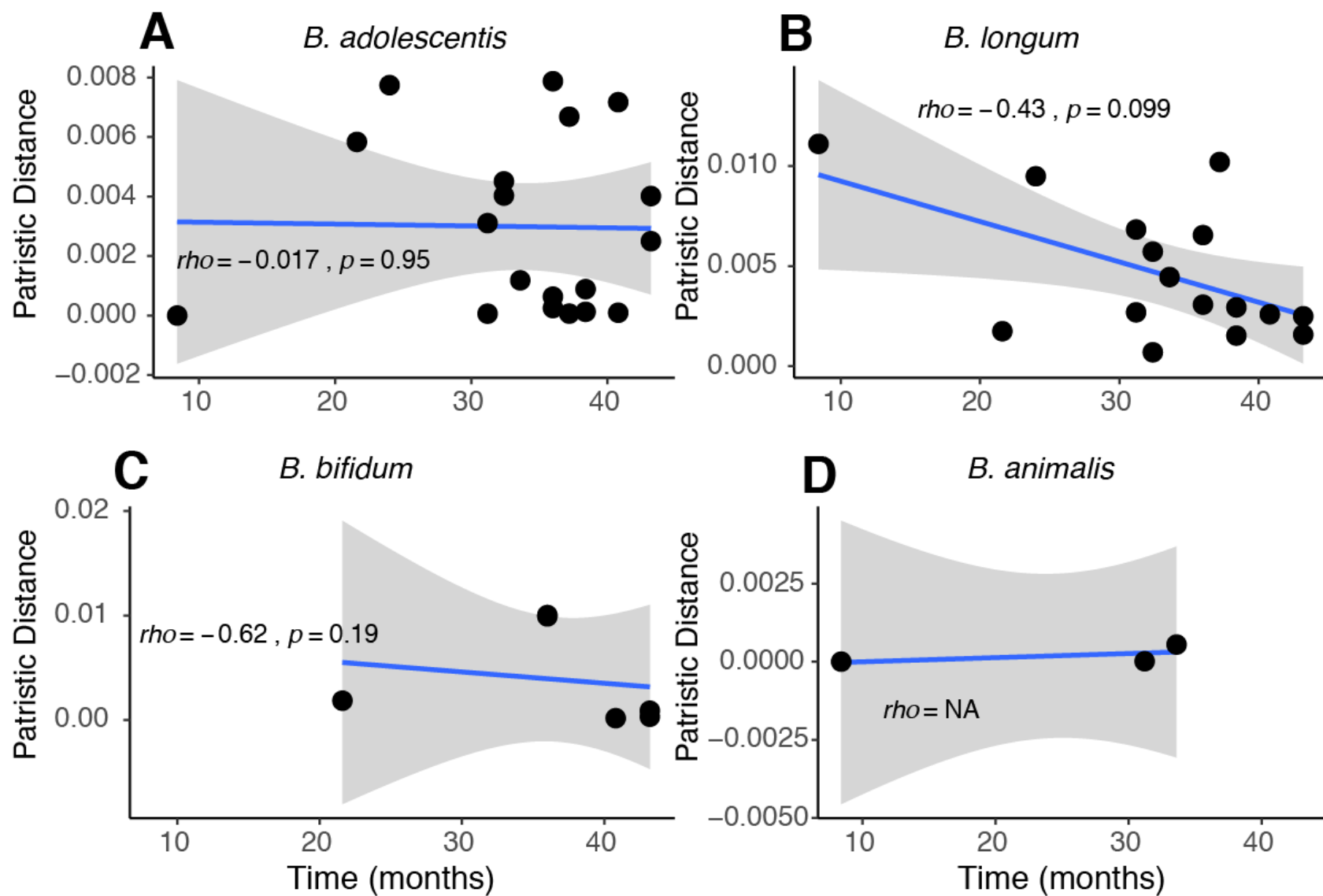

Supplement: FIG S4 [file mSystems.00911-20-sf004.pdf]

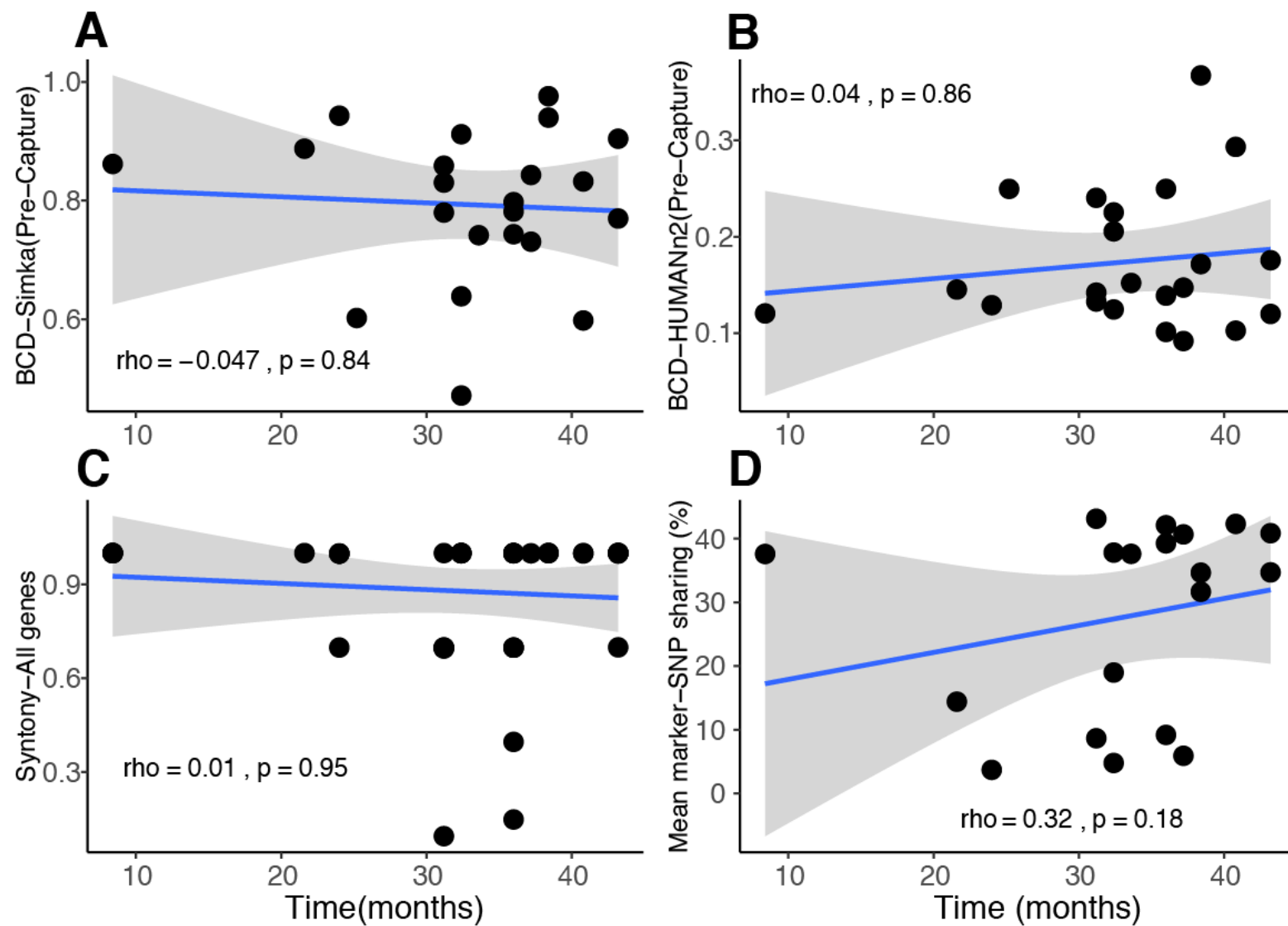

Supplement: FIG S5 [file mSystems.00911-20-sf005.pdf]

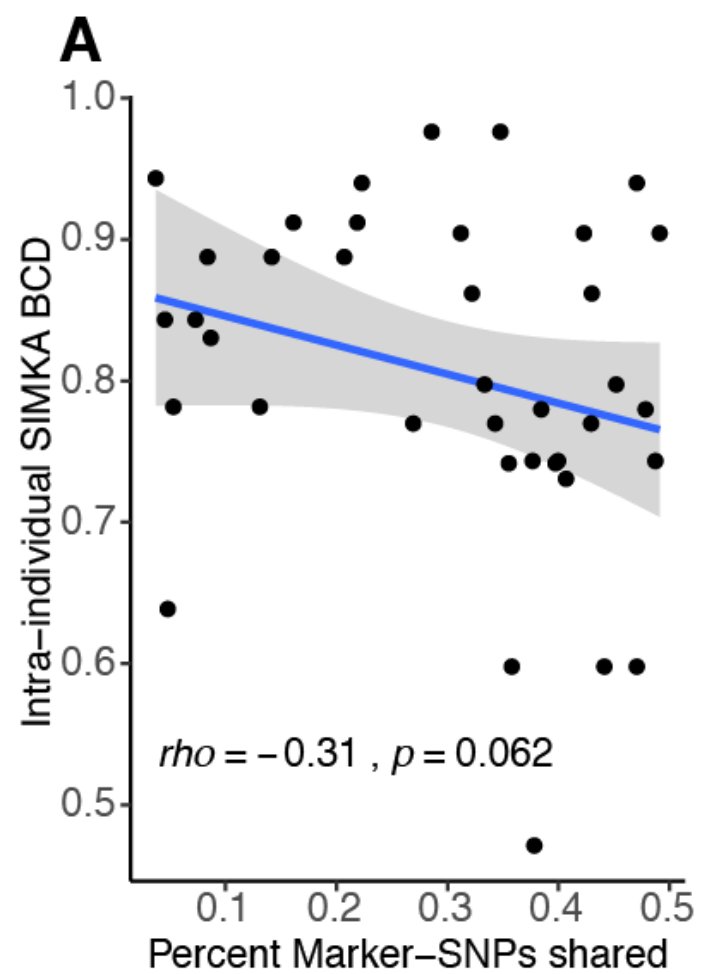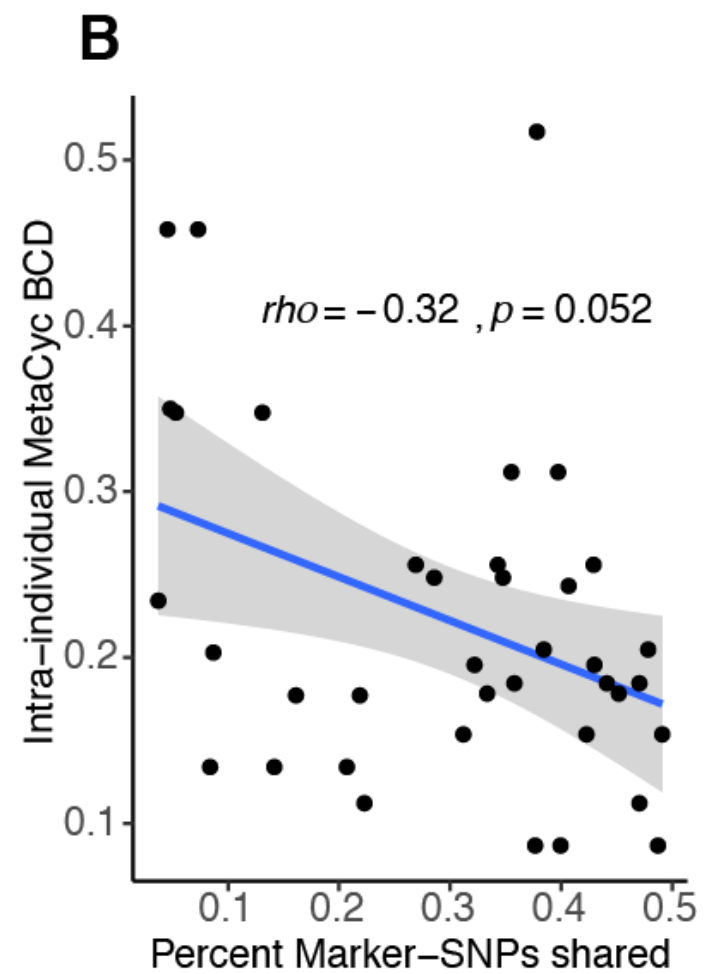

Supplement: FIG S6 [file mSystems.00911-20-sf006.pdf]

*nupG*

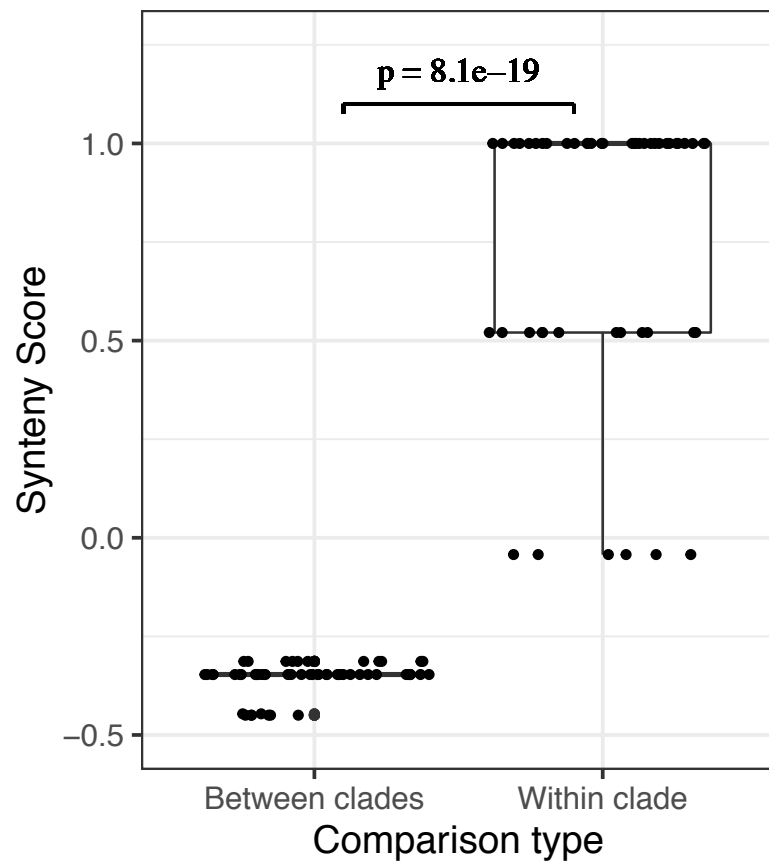

*polB*

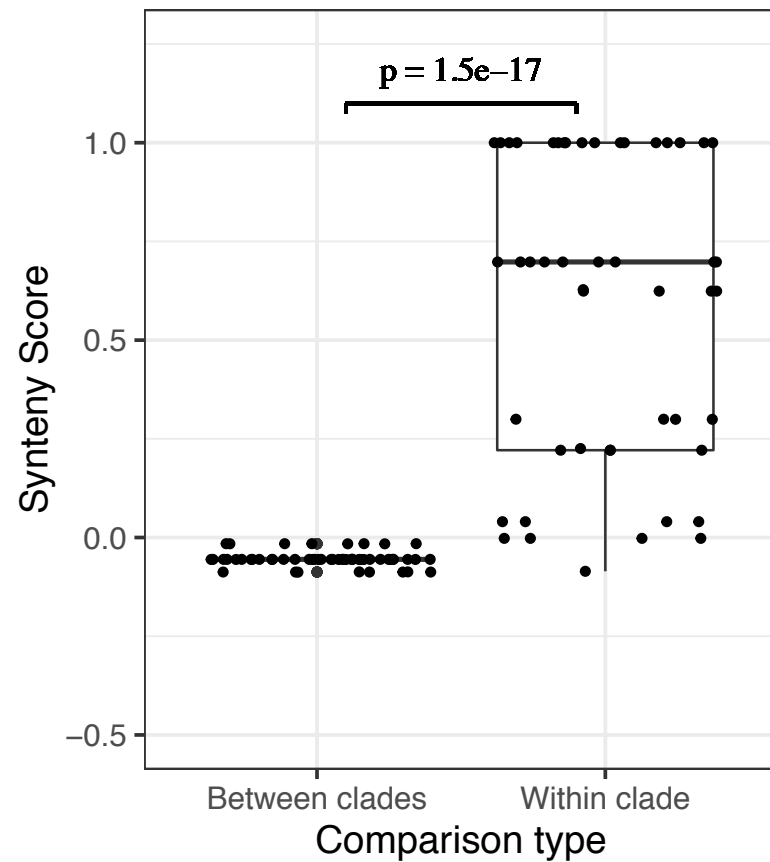

*hemA*

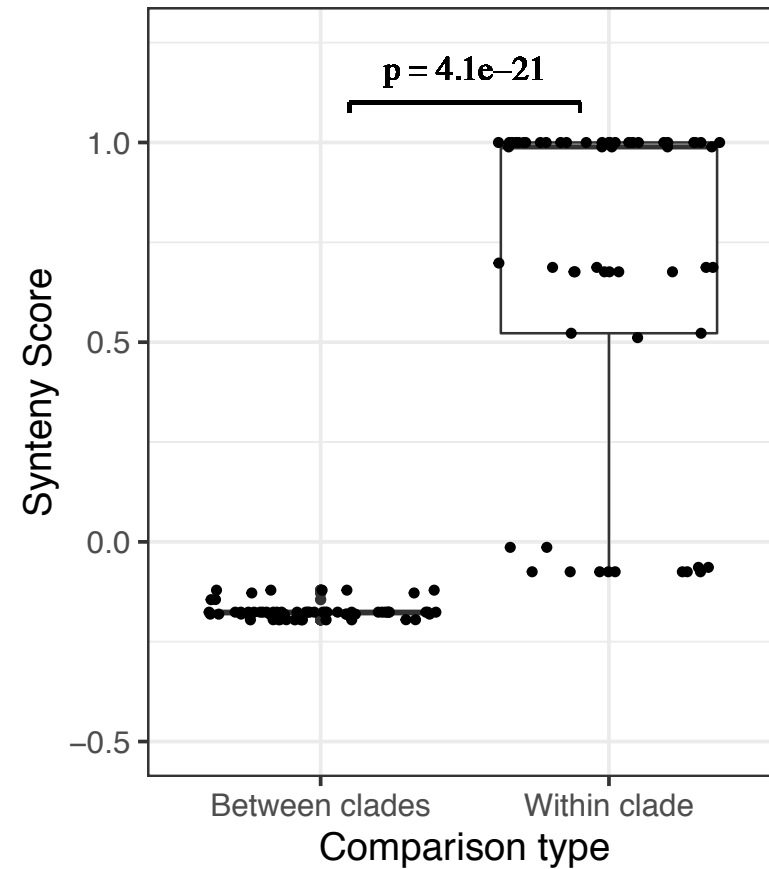

Supplement: FIG S7 [file mSystems.00911-20-sf007.pdf]
